# The Influence of Compatibility on the Structure and Properties of PLA/Lignin Biocomposites by Chemical Modification

**DOI:** 10.3390/polym12010056

**Published:** 2019-12-31

**Authors:** Jianbing Guo, Xiaolang Chen, Jian Wang, Yong He, Haibo Xie, Qiang Zheng

**Affiliations:** 1 Department of Polymer Material and Engineering, College of Materials and Metallurgy, Guizhou University, Guiyang 550025, China; guojianbing_1015@126.com (J.G.); wangjian0319jian@163.com (J.W.); hy18798074906@163.com (Y.H.); 2 National Engineering Research Center for Compounding and Modification of Polymer Materials, Guiyang 550014, China; 3 Key Laboratory of Advanced Materials Technology Ministry of Education, School of Materials Science and Engineering, Southwest Jiaotong University, Chengdu 610031, China; chenxl612@sina.com; 4 Department of Polymer Science and Engineering, Zhejiang University, Hangzhou 310027, China

**Keywords:** acetoacetate lignin, poly(lactic acid), miscibility, properties

## Abstract

Lignin, a natural amorphous three-dimensional aromatic polymer, is investigated as an appropriate filler for biocomposites. The chemical modification of firsthand lignin is an effective pathway to accomplish acetoacetate functional groups replacing polar hydroxyl (–OH) groups, which capacitates lignin to possess better miscibility with poly(lactic acid) (PLA), compared with acidified lignin (Ac-lignin) and butyric lignin (By-lignin), for the sake of blending with poly(lactic acid) (PLA) to constitute a new biopolymer based composites. Generally speaking, the characterization of all PLA composites has been performed taking advantage of Fourier transform infrared (FTIR), scanning electron microscopy (SEM), dynamic Mechanical analysis (DMA), differential scanning calorimeter (DSC), thermogravimetric analysis (TGA), rheological analysis, and tensile test. Visibly, it is significant to highlight that the existence of acetoacetate functional groups enhances the miscibility, interfacial compatibility, and interface interaction between acetoacetate lignin (At-lignin) and PLA. Identical conclusions were obtained in this study where PLA/At-lignin biocomposites furthest maintain the tensile strength of pure PLA.

## 1. Introduction

Climate change and the depletion of petroleum-based reserves are two major global challenges that are motivating research on the growing sustainability [1], and environmental-friendly and renewable resources to replace materials obtained from non-renewable fossil-based reserves in order to solve the increasing fuel demands and growing concern for the effects of greenhouse gases emissions from fossil fuels [2,3]. Biomaterials, characterized by biocompatibility and biodegradability, are potential materials in order to satisfy the requirements of sustainable development and quality of life. Hence, a large quantity of research has been dedicated to the development of biobased polymers and biocomposites from natural sources owing to their low cost, biocompatibility, and biodegradability [4].

Lignin, a natural amorphous three-dimensional aromatic polymer [5,6], consisting of methoxylated basic phenyl propanol structures [7], is considered the most promising renewable resource to petroleum-based polymer materials [8], conferring rigidity, strength, and structural support to biomass. Extensive research into the abundance and the renewable character of lignin, engendered in million tons per year as a by-product during the pulping process, has urged the potential for oil substituting raw material for the manufacture of new biobased materials [9,10]. As one of the main components of this biomass, lignin presents an aromatic backbone, making it the most important renewable source of aromatic structures, elucidating the intensive research activities worldwide. In their native forms, lignin appears to be complex, apparently unordered structures, where the different units, involving coumaryl, coniferyl, and sinapyl [11], are connected by ethers or condensed C–C bonds [12,13]. In recent years, the development of high-performance lignin-based functional materials has attracted comprehensive attention, possessing properties such as antibacterial, antioxidant, or UV-absorbance [14]. However, the lignin reactivity is supposed to be restricted as a consequence of stearic hindrance which results from the complexity and disorder of molecular structure. Hence, chemical modification seems to be a key strategy to further implement lignin into the polymer chemistry owing to abundant functional groups, such as phenolic and aliphatic hydroxyl, carbonyl, alkyl aryl ether, biphenyl, diaryl ether, etc. [15]. In particular, aliphatic and phenolic hydroxyl (OH) groups can be easily modified through esterification or alkylation, either to chain extend or introduce new chemically active sites to improve the current low degree of lignin utilization in the chemical industry and make the process economically profitable [16]. Based on its structural complexity, lignin is deemed as a fascinating filler material.

The poly(lactic acid) (PLA), identified as an eco-friendly biobased polymer for a large number of industrial products owing to good mechanical properties [17], high degree of transparency, and ease of fabrication, low emission, low energy processability, biodegradability, and recyclability [18,19,20,21], has been the subject of special attention on blending with lignin to acquire biodegradability composites applied to enormous potential and promising prospects, such as automotive components, the electrical industry, building materials, and the aerospace industry [22,23]. Naturally, the source of PLA is a green pathway to obtain, and the raw materials mainly come from renewable resources [24]. Due to its easy melting processability below the degradation temperature of plenty of natural materials, PLA is considered to be the development of totally biobased materials. Due to its superiority, PLA is prior to other products, therefore it is chosen as the first biobased commodity plastic to be produced. Nevertheless, it has inherent disadvantages including high cost and low toughness which render limitation in wide-scale adoption of polymers [25,26]. Based on the concept of sustainable development, the addition of fillers to form new-style green composites is an effective way to improve upon large-scale application.

To the best of our knowledge, lignin in combination with acetoacetate functional groups is less described [27]. Additionally, the preparation and synthesis method of acetoacetate lignin is a new methodology to form the unique structure of functional groups, with the purpose of enhancing the reactivity and compatibility of initial lignin. In this work, the effect of the strong hydrogen bond donor group on the structure and properties of polymer/lignin biocomposites as well as the compatibility between components is discussed and clarified through the chemical modification of lignin. In addition, the compounding and compatibilizing mechanism of ketone derivatives of lignin and polymer matrix were also investigated and discussed further. The aim of this work was focused on the well-known interaction between lignin and PLA to obtain novel biobased composites establishing the example of lignin-based PLA biocomposites. It is thus the objective to characterize the rheological, morphological, dynamic mechanical, tensile strength, and thermal properties of PLA, PLA/acidified lignin (PLA/Ac-lignin), PLA/acetoacetate lignin (PLA/At-lignin), and PLA/butyric lignin (PLA/By-lignin) biocomposites to elaborate miscibility, interfacial compatibility, and interface interaction between lignin filler and PLA matrix.

## 2. Materials and Methods

### 2.1. Materials

Poly(lactic acid) (PLA 4032D) with a weight-average molecular weight (*M*_w_) and a polydispersity index of 17.62 × 10^4^ g/mol and 2.1 was procured from Natureworks (Blair, NE, USA). Kraft lignin used for the composites was obtained from (Shandong Tranlin Group, Qingdao, China) using the LignoBoost process, which is extraction of lignin from black liquor.

**Scheme 1:**
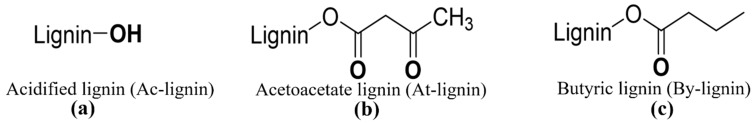
The structures of acidified lignin (Ac-lignin) (**a**), acetoacetate lignin (At-lignin) (**b**), and butyric lignin (By-lignin) (**c**).

The PLA biocomposites with three different lignin compositions were fabricated by using acidified lignin (Ac-lignin), acetoacetate lignin (At-lignin), and butyric lignin (By-lignin). The structures of Ac-lignin, At-lignin, and By-lignin are shown in Scheme 1. Table 1 highlights the naming convention used in this work based on the composition of the biocomposites.

### 2.2. Preparation of Samples

Ac-lignin: The solution of 67 g deionized water and 33 g Kraft lignin was stirred under ambient temperature for 3 h to achieve full dissolution of lignin. After centrifugation at 10,000 rpm for 5 min, the solid residue was discarded and the supernatants were collected. The addition of dilute H_2_SO_4_ (33%) dropwise into supernatants was to adjust pH to 2 or 3. After centrifugation at 10,000 rpm for 5 min, the solid residue was collected and washed thoroughly with deionized water before being freeze-dried.

At-lignin: Ac-lignin (0.5 g) was dissolved in 9.5 g Gamma-Valerolactone (GVL) to form a homogeneous solution with magnetic stirring. Then, the solution was stirred at 140 °C for 5 h to activate lignin. Next, 0.955 mL of acetyl ketene was added dropwise and stirred at 90 °C for 1 h. The crude reaction solution was added dropwise and precipitated in enthanol, and At-lignin was obtained by centrifugation and washed thoroughly with deionized water before free-drying [28].

By-lignin: Samples of Ac-lignin were placed in a 100 mL round-bottomed flask to which the butyric anhydride (25 mL) and 1-methylimidazole (1-MIM) (0.5 mL) were added. The reaction was kept at 120 °C for 24 h with efficient stirring. The By-lignin was recovered by adding the reaction mixture dropwise into water with stirring to allow for complete precipitation, followed by centrifugation, and washed exhaustively with deionized water before free-drying [29].

**Figure 1:**
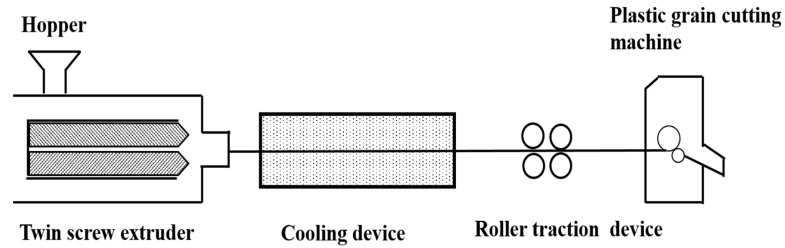
The melt-impregnation process of PLA/lignin biocomposites.

The preparation of PLA/lignin biocomposites: The pure PLA was first blended with different lignin as the ratio listed in Table 1, and dried in an oven at 80 °C for 8 h to remove moisture. As shown in Figure 1, the PLA/lignin master batches were prepared by using a micro twin-screw extruder (SJZA-10A, Ruiming Experiment Apparatus Manufacturing Co., Ltd., Wuhan, China) at the processing temperature from 160 °C to 180 °C. Then they were injection molded into standard specimens at 180–200 °C by the micro injection molding machine (type of SZS-20, Ruiming Experiment Apparatus Manufacturing Co., Ltd., Wuhan, China).

### 2.3. Measurements and Characterization

#### 2.3.1. Fourier-Transform Infrared (FTIR) Spectroscopy

FTIR spectra were recorded on a NEXUS-570 spectrophotometer (Thermo Fisher Scientific Co., Ltd., Waltham, MA, USA) in a range of wavenumber from 4000 to 500 cm^−1^ with the resolution and scanning number of 4 cm^−1^ and 32 times, respectively. The samples for FT-IR analysis were prepared by pressing with KBr at a mass proportion of about 1/100.

#### 2.3.2. NMR Spectroscopy

In the present work, the ^31^P NMR analysis was performed on lignin samples phosphytilated with 2-chloro-4,4,5,5-tetramethyl-1,3,2-dioxaphospholane, using cholesterol as internal standard, according to standard protocol. Then, 128 scans were recorded with a 15 s delay and a spectral width of 80 ppm (180–100 ppm). For quantitative ^1^H NMR analysis, 20 mg of lignin was dissolved in 0.5 mL of DMSO-d_6_. All NMR spectra were recorded on a Bruker Ascend 400 MHz spectrometer (Bruker Corporation, Karlsruhe, Germany). Then, 32 scans were collected with a 10 s delay. The ^13^C-NMR spectra were recorded on a Bruker Ascend 400 MHz and an inverse-gated decoupling sequence with a relaxation time of 4 s. The number of scans was at least 10,000. The samples were dissolved in DMSO-d_6_ and measured at 60 °C.

#### 2.3.3. Scanning Electron Microscopy (SEM)

For viewing the morphology of the samples, the samples obtained upon fabrication were freeze-fractured inside liquid nitrogen. The fractured surface was then sputter coated. The impact fracture surfaces of the PLA/lignin samples were observed by a KYKY-2800B SEM (KYKY Technology Development Ltd., Beijing, China) equipment with an acceleration voltage of 25 kV. SEM images of all the samples were recorded after the surfaces of the samples were coated with gold.

#### 2.3.4. Atomic Force Microscope (AFM)

AFM experiments were performed at room temperature using a Dimension Icon Atomic Force Microscope (Bruker Corporation, Karlsruhe, Germany) operating in tapping mode and peak force tapping with amplitude control at 1.0 Hz. The data were analyzed using the Nano Scope software version 1.40 (Bruker Corporation, Karlsruhe, Germany). Scan sizes were 5–10 μm with 512 scans on each scanning line.

#### 2.3.5. Dynamic Mechanical Test

The rectangular biocomposites obtained upon fabrication were tested in a dynamic mechanical analyzer (Q800, TA Co., Ltd., New Castle, PA, USA) using a dual cantilever setup. For obtaining the dynamic mechanical properties, a constant displacement amplitude-isothermal frequency ramp procedure was used on all samples. In this procedure, a central vertical displacement amplitude of 15 μm, at a temperature of 30 °C, using a frequency sweep from 5 to 45 Hz was specified.

#### 2.3.6. Differential Scanning Calorimetry (DSC)

About 8 mg of the specimen to be tested was weighed and hermetically sealed in aluminum pans. The spans were then placed in sequence in an auto sampler of a DSC analyzer (Q10, TA Instruments, TA Co., Ltd., New Castle, PA, USA) for testing. A heat-cool-heat program was used for all samples. To capture the heat flow behavior with rate of temperature change, three different heating/cooling rates were used. For each heating/cooling rate, the samples were first heated to 200 °C, held 5 min to erase the thermal history, then cooled to 30 °C and reheated to 200 °C.

#### 2.3.7. Thermogravimetric Analysis (TGA)

For each test, between 10 and 15 mg of material to be tested was placed in a platinum pan and heated in the chamber of a thermogravimetric analyzer (Q50, TA Co., Ltd., New Castle, PA, USA). The chamber containing the sample was continuously purged with nitrogen gas at a flow rate of 10 mL/min. All samples were heated to 800 °C using a heating rate of 10 °C/min.

#### 2.3.8. Rheological Analysis

The rheological properties were measured using a TA rheometer (AR 2000, TA Co., Ltd., New Castle, PA, USA) with a nitrogen purge. A strain amplitude of 1% was found to be suitable to ensure linear viscoelastic regime and thus used for both the frequency sweep and temperature ramp sweep modes.

#### 2.3.9. Mechanical Properties

The tensile tests were measured with an electronic universal mechanical testing machine (WDW-10C, Shanghai Hualong Test Instrument Co., Ltd., Shanghai, China) according to the ASTM D-638. The experiment was carried out at room temperature with a cross-head speed of 10 mm/min. The gauge length between the two pneumatic clamps was 25 mm. Five measurements were performed for each sample, and the averaged result was reported.

## 3. Results and Discussion

### 3.1. The Structure and Morphology of Lignin and Its Biocomposites

**Figure 2:**
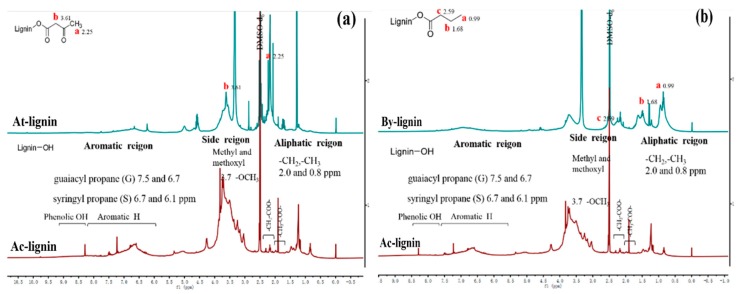
The ^1^H NMR spectra of (**a**) Ac-lignin and At-lignin; (**b**) Ac-lignin and By-lignin.

In view of ^1^H NMR spectra shown in Figure 2, the signals at 6.7–6.1 and 7.5–6.7 ppm were attributed to aromatic protons in syringyl-propane and guaiacyl-propane structures [30], and the Hα in β-O-4 structures exhibited single signal at 6.1–5.8. Distinctly, it was found that the Hα in β-5 structures existed at 5.8–5.2, Hα and Hβ in β-O-4 structures showed signal peaks at 4.9–4.3, and Hα in β-β structures appeared at 4.3–4.0. Moreover, methoxyl protons (–OCH_3_) gave a sharp signal at 3.61 ppm. By observation and comparison, it was discovered clearly that H protons in aromatic rings got weak compared to initial acidified lignin, demonstrating the existence of new chemical structure. In Figure 2a, H protons in methyl (–CH_3_) and methylene (–CH_2_–) appeared in 3.61 and 2.25, respectively, which were ascribed to acetoacetate groups [31]. Accordingly, H protons in methylene (–CH_2_–) existed at 2.59 and 1.68 ppm, respectively, and H protons in methyl (–CH_3_) appeared at 0.99, which were attributed to the existence of new chemical structure, as shown in Figure 2b.

**Figure 3:**
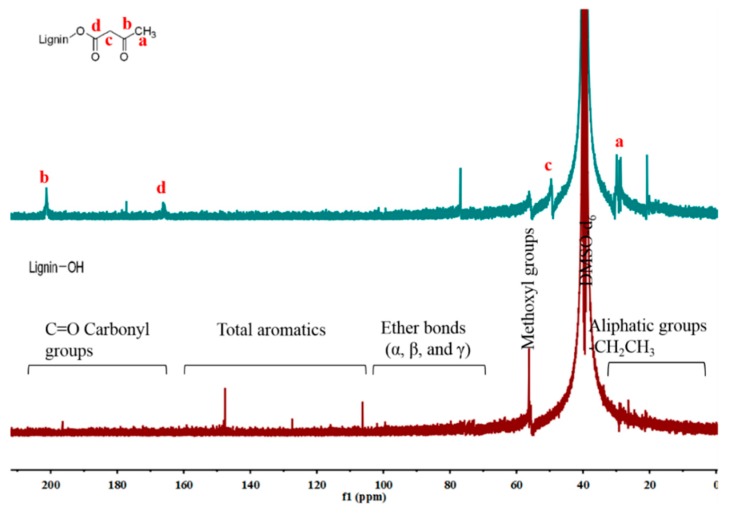
The ^13^C NMR spectra of Ac-lignin, At-lignin, and By-lignin.

**Figure 4:**
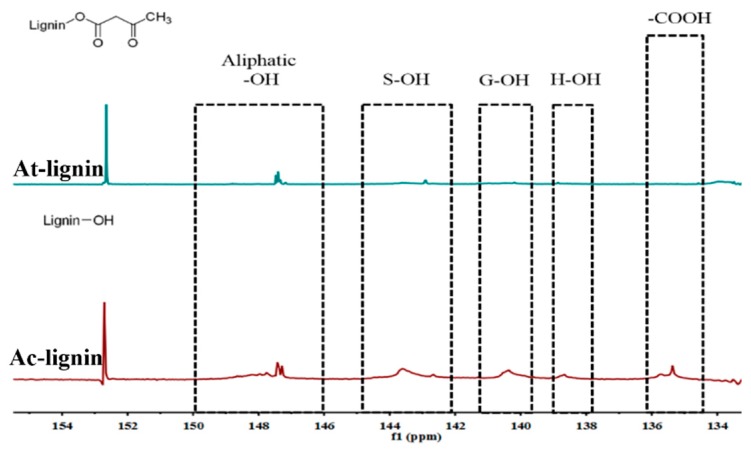
The ^31^P NMR spectra of Ac-lignin and At-lignin.

The ^13^C NMR was used to further investigate the carbonate structures of lignin formed [31,32]. In general, the spectrum of different lignin can be divided into the region of aromatic (100–160 ppm) and aliphatic (10–90 ppm) carbon atoms, as shown in Figure 3. Compared with initial lignin, it was discovered that the successful acetoacetate lignin was verified by the signals of new acetoacetate groups (a: 30.3 ppm, b 200.4 ppm, c 50.7 ppm, d 167.9 ppm). In addition, the signals of carboxyl groups (C=O) occurred from 165–210 ppm, and the signal at around 70–105 ppm was attributed to the ether bonds [31]. By the comparison of spectra, the signal at about 20–30 ppm showed a significant intensity increase, which was ascribed to carbon atoms of aliphatic chains. The ^31^P NMR spectroscopy proved to be a useful tool to study the structures of lignin [33]. Naturally, the different types of active OH groups (aromatic, aliphatic, and carboxylic) and the S/G/H ratio in lignin were evaluated to further investigate the structure of lignin. The spectra shown in Figure 4 presented resonances assigned to various units bearing free OH groups: Aliphatic OH groups (broad signal at 145–150 ppm), phenolic OH groups (at 141.5–144 ppm for S, at 139–140 ppm for G, and at 137–138.5 ppm for H), and carboxylic acid groups (at 136.2–134.5 ppm) [33]. Obviously, it was observed that the signals of S, G, H, and carboxylic acid groups in acetoacetate lignin decreased compared with initial lignin, expressing that most OH groups were consumed by chemical modification. In view of reference, the synthesis of By-lignin was successful and the functional groups were grafted.

**Figure 5:**
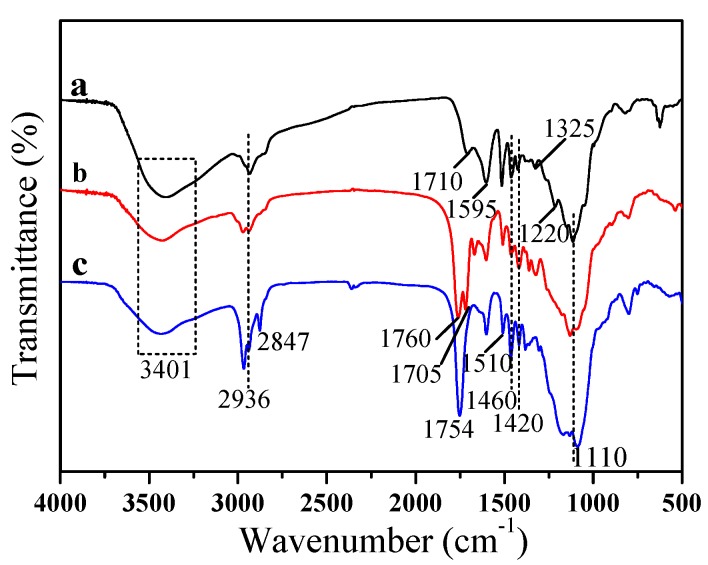
FTIR spectra of Ac-lignin (**a**), At-lignin (**b**), and By-lignin (**c**).

**Figure 6:**
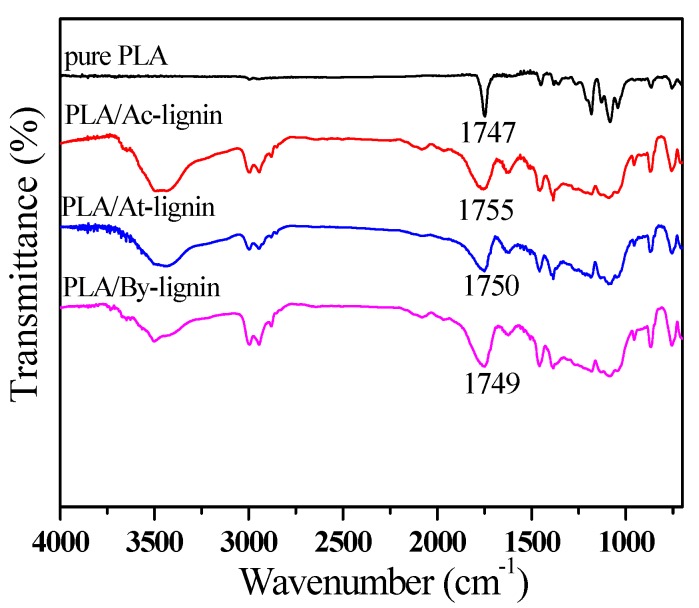
FTIR spectra of pure PLA, PLA/Ac-lignin, PLA/At-lignin, and PLA/By-lignin.

FTIR spectra of Ac-lignin, At-lignin, and By-lignin are shown in Figure 5. In the case of Ac-lignin as shown in Figure 5a, the wide absorption band at 3401 cm^−1^ originated from the presence of O–H stretching vibrations in aromatic and aliphatic O–H groups. Whereas the bands at 2936 and 2847 cm^−1^ were attributed to the C–H asymmetric and symmetric vibrations in the methyl and methylene groups [33]. The band at 1710 cm^−1^ shows the presence of nonconjugated carboxylic acids. The peaks at 1595 and 1510 cm^−1^ were ascribed to C–C of aromatic skeletal vibrations. It was found that the bands at 1460 and 1420 cm^−1^ were imputed to the C–H deformation in –CH_2_– and –CH_3_ groups and C–H aromatic ring vibrations, respectively [34]. At the same time, it was detected that some characteristic bands associated to syringyl and guaiacyl units in lignin were found at 1325, 1220, and 1110 cm^−1^ corresponding to syringyl and condensed guaiacyl absorptions, guaiacyl ring breathing, C–C, C–O stretch, and aromatic C–H in plane deformation. In the case of At-lignin and By-lignin (Figure 5b,c), it is obvious that the signal around 3401 cm^−1^ which corresponded to OH stretching vibrations in aromatic and aliphatic OH groups was impaired compared with Ac-lignin. Obviously, the appearance of two peaks at 1760 and 1705 cm^−1^ in At-lignin was clearly seen, which was assigned to acetoacetate groups. This indicates that acetoacetate process was successful, and the appearance of peak at 1710 cm^−1^ shifting to 1754 cm^−1^ showed the change of carboxyl group, manifesting the success of acetylation process. Figure 6 shows the FTIR of PLA, PLA/Ac-lignin, PLA/At-lignin, and PLA/By-lignin. In view of PLA, C–O bond at 1747 shifted to 1755, 1750, and 1749 cm^−1^ in PLA/Ac-lignin, PLA/At-lignin, and PLA/By-lignin, which was attributed to the results of the hydrogen bond between lignin and PLA molecule. In addition, for different lignin derivatives, the shifting degree of C–O of three biocomposites was different. This indicated also the addition of lignin had an influence on the properties of PLA biocomposites owing to the degree of miscibility and interfacial adhesion between PLA and different lignin.

**Figure 7:**
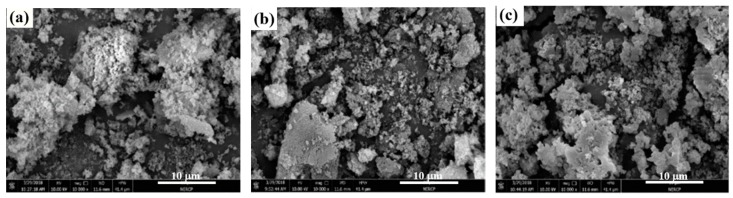
SEM photos of (**a**) Ac-lignin, (**b**) At-lignin, (**c**) By-lignin.

Figure 7 shows the SEM micrographs of the acidified lignin, acetoacetate lignin, and butyric lignin. There was a scatter in the size ranging from 0.5 m to 2 m for the Ac-lignin, At-lignin, and By-lignin; but, the particle size of At-lignin was much smaller than that of Ac-lignin and By-lignin. The particle shape of lignin was important to affect the degree of anisotropy. It can be seen clearly that all particles of lignin appeared to be roughly spherical, indicating that different level accumulation of lignin particles led to the occurrence of various agglomerates. At the same time, the lignin size also affected the surface area to volume ratio, resulting in differences in stress transfer and matrix-particle filler wetting, further adjusting and regulating the compatibility and consistency between lignin filler and PLA matrix.

**Figure 8:**
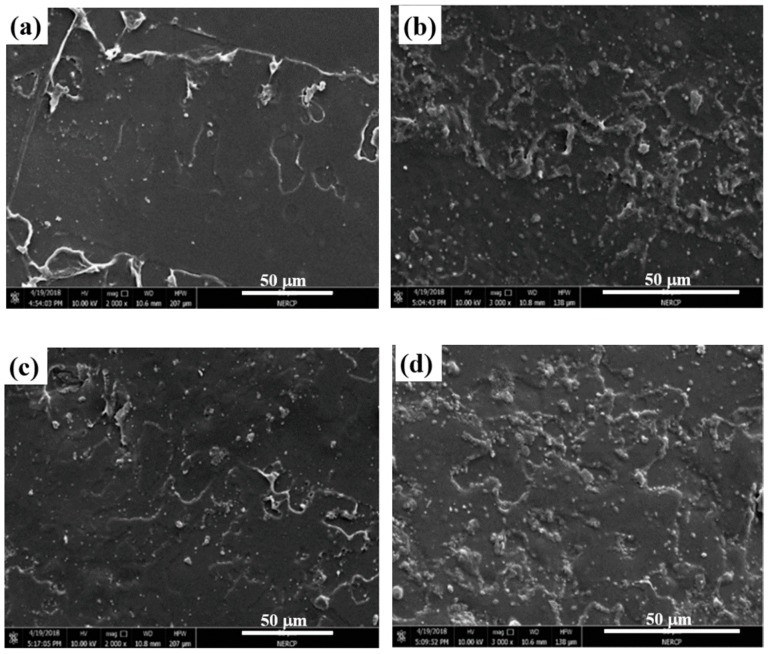
SEM micrographs of PLA and its biocomposites: (**a**) PLA, (**b**) PLA/Ac-lignin, (**c**) PLA/At-lignin, (**d**) PLA/By-lignin.

The cross-sectional morphologies of pure PLA and PLA/lignin biocomposites are shown in Figure 8. It is evident that the increase of heterogeneity of the blends resulting in varieties of particle sizes was attributed to the incorporation of lignin into the pure PLA, which gave rise to greater areas of weakness within the biocomposites, thereby possibly reducing its tensile strength. On the strength of slightly greater various particle sizes affected with the degree of miscibility and interfacial adhesion between PLA and different lignin, it was found that Ac-lignin, At-lignin, and By-lignin uniformly dispersed and embedded in the PLA matrix [35]. Obviously, it was visible to observe that the surface of PLA/lignin biocomposites was rougher than the pure PLA, embracing spheroidal-like lignin particles embedded within the matrix [36]. With the addition of At-lignin, a distinct feature, which can easily be seen in Figure 8c, is that distinct phase is still visible with distinguishable lignin particles compared with Figure 8b,d. It can be observed that a large number of lignin particles were protruded out of the surface, indicating that there was a poor compatibility between PLA and Ac-lignin or By-lignin compared with PLA and At-lignin in PLA/At-lignin biocomposites. In other words, the incorporation of At-lignin was more beneficial to improve the dispersion in the filler phase with matrix PLA [37], thus confirming the better interaction and adhesion between PLA and At-lignin, which contributed to form a stronger matrix-filler interface [38]. A point to note from the SEM micrographs is that PLA/At-lignin biocomposites showed better properties than PLA/Ac-lignin and PLA/By-lignin biocomposites due to the stronger interfacial interaction of matrix filler.

**Figure 9:**
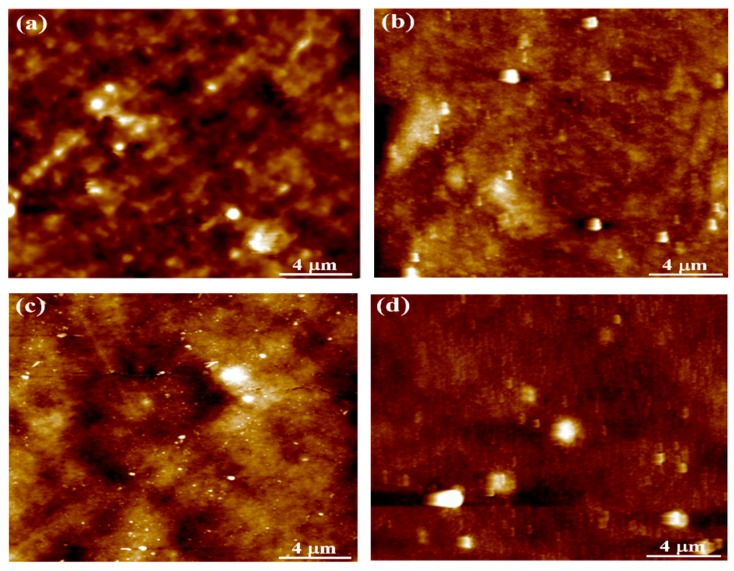
AFM height images of the surface of PLA and its biocomposites: (**a**) PLA, (**b**) PLA/Ac-lignin, (**c**) PLA/At-lignin, (**d**) PLA/By-lignin.

**Figure 10:**
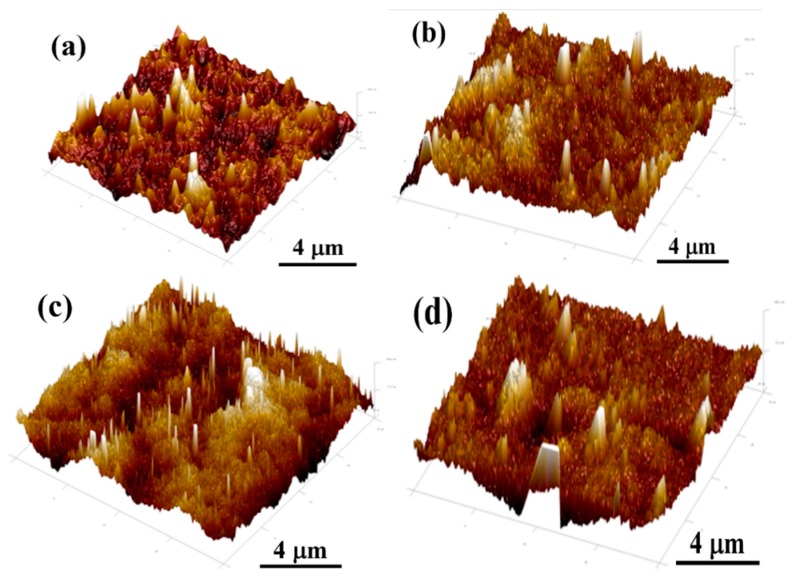
The 3D AFM micrographs of the surface of PLA and its biocomposites: (**a**) PLA, (**b**) PLA/Ac-lignin, (**c**) PLA/At-lignin, (**d**) PLA/By-lignin.

The AFM analysis was also performed to further measure the surface morphologies and dispersion of polymer and its blends [39,40]. Figure 9 and Figure 10 exhibit the tapping images of PLA, PLA/Ac-lignin, PLA/At-lignin, and PLA/By-lignin biocomposites. It is evident from Figure 9 and Figure 10 that the surface of pure PLA and PLA/lignin biocomposites showed a significantly different roughness behavior, and, depending on the different modified lignin, the structure on the surface of the samples varied greatly. For example, the surface area difference of PLA is 0.169%. However, the values of surface area difference of PLA/Ac-lignin, PLA/At-lignin, and PLA/By-lignin increased to 0.470%, 1.21%, and 0.476%, respectively. It is obvious that PLA/At-lignin biocomposites had the most surface roughness compared with other samples, as shown in Figure 9c and Figure 10c. In addition, after chemical modification, the chain movement at the surface increased significantly, which led to the formation of more ordered structures. On the one hand, the PLA/At-lignin sample modified with acetoacetate lignin exhibited a miscible biocomposite due to the enhanced interfacial adhesion between PLA matrix and lignin filler compared with the other two biocomposites. In fact, similar work has been reported by Teramoto et al. [41]. The blend of untreated lignin exhibited a phase separation. However, the blends with modified lignin (by using butyric and valeric anhydrides) presented good miscible blends and an absence of phase separation [41]. On the other hand, it was clearly found that the dispersion of At-lignin in the PLA matrix was improved.

### 3.2. Dynamic Mechanical Analysis of the Biocomposites

Storage modulus (*E*′) was obtained from the strain response in phase with the stress and was a measure of the strain energy stored elastically. The loss modulus (*E″*) was obtained by the out of phase strain response and represented strain energy lost in viscous dissipation per cycle. The ratio of the *E″* to *E*′ represents tanδ which is a measure of the proportion of input energy dissipated. The following Equations (1)–(3) describe the relation between the above mentioned viscoelastic properties to the stress input and strain response of the material [42]:

(1) $$E^{\prime }=\frac{\sigma_{0}\left(t\right)}{\varepsilon \left(t\right)}=\frac{\sigma_{0}}{\varepsilon_{0}}\cos\left(\delta \right)$$

(2) $$E^{\prime \prime }=\frac{\sigma_{\pi -0}\left(t\right)}{\left(\frac{\varepsilon \left(t\right)}{\omega }\right)}=\frac{\sigma_{0}}{\varepsilon_{0}}\sin\left(\delta \right)$$

(3) $$\tan\delta =\frac{\sigma_{\pi -0}\left(t\right)}{\left(\frac{\varepsilon \left(t\right)}{\omega }\right)}$$

where ε(t) is the applied strain, ε(t) is the applied strain rate, ε_0_ is the applied strain amplitude, σ_0_(t) is the stress in phase with the strain, σ_π-0_(t) is the stress out of phase with the strain, σ_0_ is the stress amplitude, and δ is the phase angle. *E*′ is the stress required per unit of elastic strain and *E″* is the stress required per unit of viscous strain. All quantities were calculated over one cycle. The *E*′ and *E″* obtained represent the responses of the spring and dashpot, respectively, in the Kelvin–Voigt model. However, the distribution of the applied strain energy between elastic and viscous components depends on the ratio of the loss modulus to the storage modulus which is defined as the loss tangent, tanδ.

**Figure 11:**
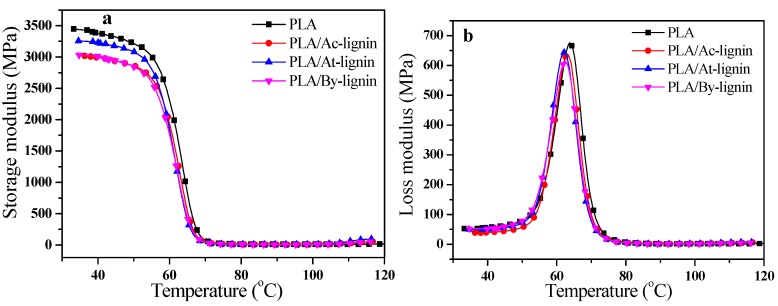

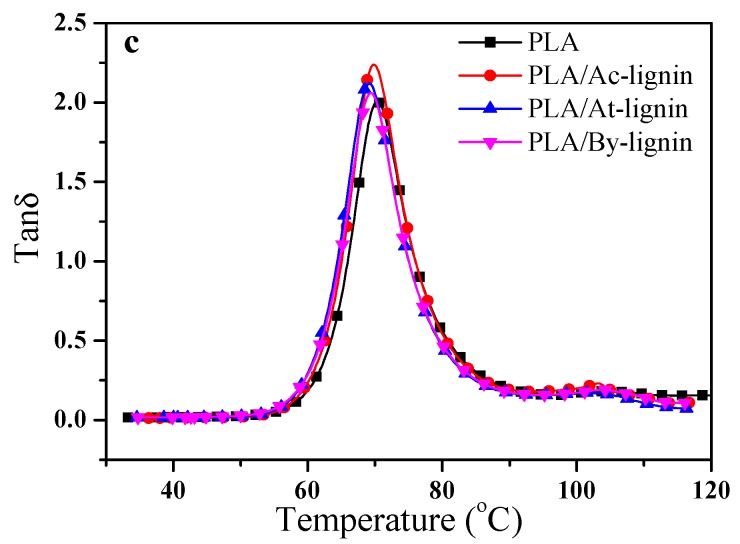
Dynamic mechanical properties of PLA and PLA-lignin biocomposites: (**a**) Storage modulus, (**b**) loss modulus, and (**c**) tanδ.

The most important finding was that the individual constituents and the interactions of matrix-filler played a significant role on the dynamic mechanical properties of the biocomposites. In addition, the adhesion between the polymer matrix and the filler is evaluated by DMA technique [43]. Figure 11 displays the *E*′, *E″*, and tanδ of pure PLA, PLA/Ac-lignin, PLA/At-lignin, and PLA/By-lignin biocomposites as a function of frequency. It is interesting to note that in most of this study the stress transfer across the interface may be susceptible to the characteristics of the interface between the matrix and filler. For one thing, there was no doubt that the weaker interfaces were supposed to generate more rotational and translational degrees of freedom along with the interface, which in turn may result in increasing energy dissipation and reducing storage of energy across the interface per unit strain through stick-slip motion. For another thing, the frictional dissipation mechanisms led to an increase for the value of *E″* and damping, owing to weaker interfaces. On the whole, the overall mechanical behaviors of the biocomposites were primarily affected by particulate volume within the biocomposites and the characteristics of the matrix-filler interface.

The *E*′ curves of PLA, PLA/Ac-lignin, PLA/At-lignin, and PLA/By-lignin biocomposites are shown in Figure 11a. Pure PLA and PLA/Lignin biocomposites showed similar behaviors that the *E*′ value gradually reduced with increasing the temperature. Otherwise, the only distinct difference was that the *E*′ of PLA/lignin biocomposites was less than that of pure PLA, which was attributed to the damping of the interfacial interaction with various lignin blended with PLA. PLA/At-lignin biocomposites embrace higher *E*′ value compared with PLA/Ac-lignin and PLA/By-lignin biocomposites as a consequence of relatively outstanding matrix-filler interface and the enhancement of interfacial adhesion, which profiles that molecular chains of PLA/At-lignin took possessed mobility from the frozen state at low temperature [44], emphasizing that PLA/At-lignin biocomposites were endowed with preferable elasticity compared with PLA/Ac-lignin and PLA/By-lignin biocomposites. Moreover, the addition of acetoacetate groups in lignin remarkably changed the structure of acidified lignin, which further improved the interaction with PLA.

The *E″* stands for the degradation or the loss of energy at heat according to the cycle of sinusoidal distortion, as shown in Figure 11b. The objective of this figure is to describe that the value of *E″* appeared to be higher peak in a certain temperature range, which signified that some kind of transformation occurred in the movement of polymer, especially the thawing of some movement. As a consequence of little addition of modified lignin, the change of *E″* was not obvious.

The maximum of tanδ is always used to evaluate the glass transition temperature (*T*_g_) of polymer composites as shown in Figure 11c. For the PLA/Ac-lignin, PLA/At-lignin, and PLA/By-lignin biocomposites, the peak value of tanδ shifted to a lower value compared with pure PLA, which implies that the mobility of the molecules was impaired, owing to the increased interfacial weakness which was derived from the capability of homogeneous particle dispersion [45]. Notably, the tanδ of PLA/At-lignin biocomposites showed relatively low value in comparison with the others, which was ascribed to the better interaction between At-lignin and PLA, inducing stronger interfacial adhesion hindering the mobility of molecules [46].

### 3.3. Crystallization Behavior of the Biocomposites

**Figure 12:**
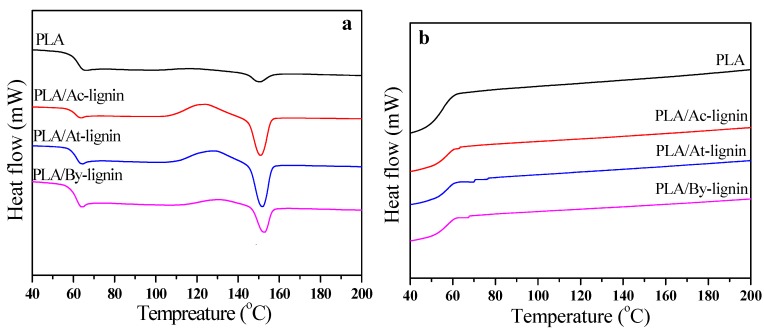
DSC thermograms of PLA and PLA/lignin biocomposites: (**a**) melting curves; (**b**) crystallization curves.

**Figure 13:**
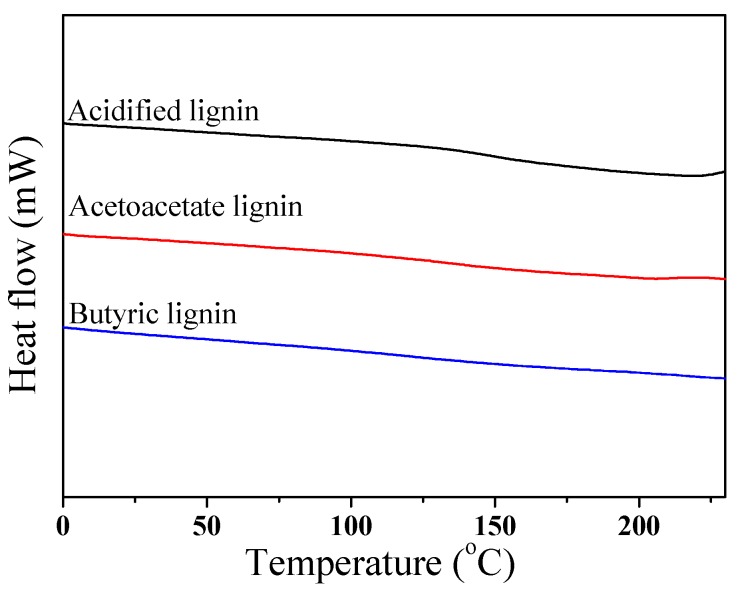
DSC thermograms of acidified, acetoacetate, and butyric lignin.

DSC is usually used to measure the heat capacity of materials [42]. Based on the *T*_g_ and melting behaviors, the interaction between the PLA and lignin phases in the biocomposites was investigated to justify the domestic relations and the miscibility between PLA and various lignin, Ac-lignin, At-lignin, and By-lignin [47]. The DSC curves of the investigated samples are demonstrated in Figure 12 and the DSC curves of three different lignin samples are shown Figure 13. Firstly, it was found that modified acetoacetate lignin and butyric lignin showed little change, compared with initial acidified lignin, further exhibiting that blending with PLA embraced different function. The glass transition behavior of PLA/Ac-lignin, PLA/At-lignin, and PLA/By-lignin biocomposites showed a sharp transition compared to that of pure PLA (listed in Table 2), which extended over a broad temperature range. A broad *T*_g_ observed for PLA/lignin biocomposites was caused by the complex molecular structure that led to multiple relaxation phases within the same transition temperature range [39]. In addition, there was a slight change in the glass transition for different lignin derivatives added. The *T*_g_ values of PLA/At-lignin and PLA/By-lignin were slightly higher than of that of PLA/Ac-lignin. This indicates the change of thermal stability of three biocomposites with different lignin derivatives, which is consistent with TGA results. Since the glass transition was a local chain relaxation phenomenon, a change in glass transition by the addition of lignin derivatives will require significant local modification of matrix mobility through the matrix-filler interfacial area. As observed in the SEM micrographs and the tensile properties, the matrix–filler interface displayed a different degree of load transfer capabilities. It is believed that the weaker matrix-filler interactions may have a contribution towards the insignificant change in the observed glass transition.

Obviously, the phase miscibility between PLA and lignin was also observed in view of the melting temperature (*T*_m_) of the crystalline phase in PLA. The *T*_m_ and the heat of melting enthalpy (Δ*H*_m_) of the biocomposites are listed in Table 2. Both *T*_m_ and Δ*H*_m_ values increased with the addition of different lignin into PLA matrix. The increase was primarily caused by the increase in chemical potential of the crystalline PLA phases as a result of molecular level miscibility with lignin [48]. It was also confirmed that the biocomposites were becoming more regular with the addition of different lignin at the same concentration.

### 3.4. Thermal Stability of the Biocomposites

**Figure 14:**
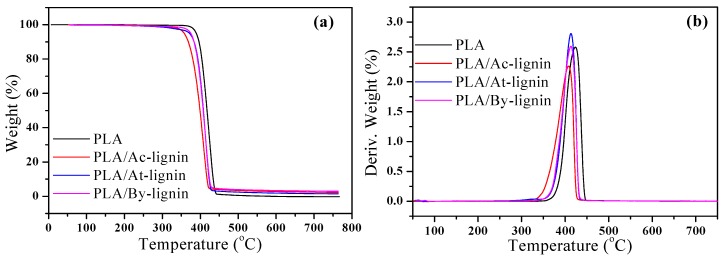
TGA (**a**) and derivative thermogravimetry (DTG) (**b**) curves of PLA and PLA/lignin biocomposites.

**Figure 15:**
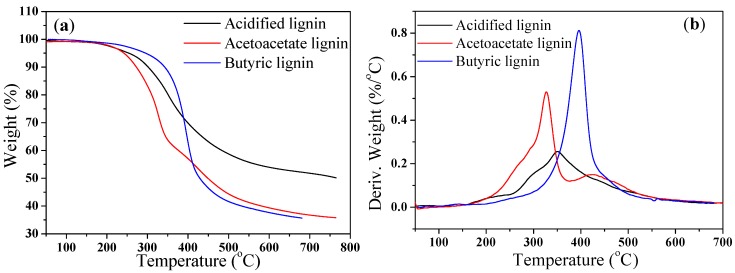
TGA (**a**) and DTG (**b**) curves of acidified, acetoacetate, and butyric lignin.

The TGA curves for PLA, PLA/Ac-lignin, PLA/At-lignin, and PLA/By-lignin biocomposites are shown in Figure 14. The temperature for the onset of maximum degradation (*T*_max_) taken from the peak temperature of the derivative wt % curves are exhibited in Table 3. Pure PLA was stable without major weight loss up to 389.4 °C, while the *T*_5%_ values of PLA/lignin biocomposites showed a continuous decrease compared with pure PLA. It was found that PLA/At-lignin and PLA/By-lignin biocomposites exhibited higher thermal stability than PLA/Ac-lignin biocomposites, which exhibited similar thermal stability of acidified, acetoacetate, and butyric lignin in Figure 15. The presence of molecular level interactions between PLA and diverse lignin resulted in the above phenomenon.

The rate of degradation of PLA/lignin biocomposites after *T*_max_ was significantly higher than that of pure PLA. Surpassing *T*_max_, the process of degradation and the volatilization of monomer occupied the main phenomenon of PLA and PLA/lignin biocomposites, while a gradual weight loss was discovered for the samples with lignin up to 500 °C, followed by constant residual weight plateau. Meanwhile, PLA/At-lignin and PLA/By-lignin biocomposites embraced higher *T*_max_ than PLA/Ac-lignin biocomposites. Obviously, it indicated that the thermal stability of PLA/At-lignin and PLA/By-lignin biocomposites was higher than that of PLA/Ac-lignin biocomposites. However, the thermal stability of all the PLA/lignin biocomposites was worse than that of pure PLA. In view of the above phenomena, the main reason is that the modified lignin took possession of stronger molecular interaction than initiative lignin due to functional groups imported lignin [49]. Generally speaking, a main and major degradation including complete decomposition of the cyclic aromatic structure of lignin in an oxygen-rich atmosphere or condensation of the same structure to carbon-rich material under inert atmosphere is expected 400 °C. In our current work, the residual weight formed above 500 °C for PLA/lignin biocomposites was primarily ascribed to the condensation of cyclic structures into carbon-rich materials compared with pure PLA. Owing to the complete volatilization of PLA, lignin was deemed as the main resource of char residues listed in Table 3.

### 3.5. Rheological Behaviors of the Biocomposites

**Figure 16:**
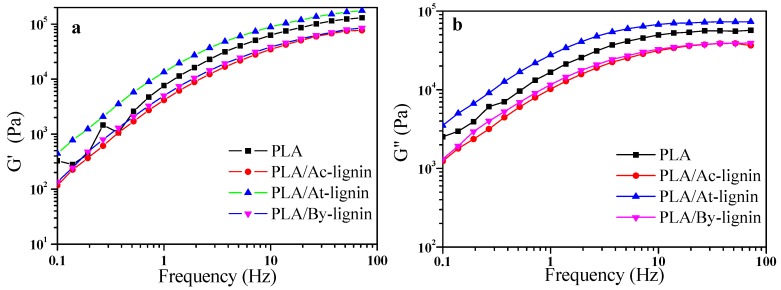

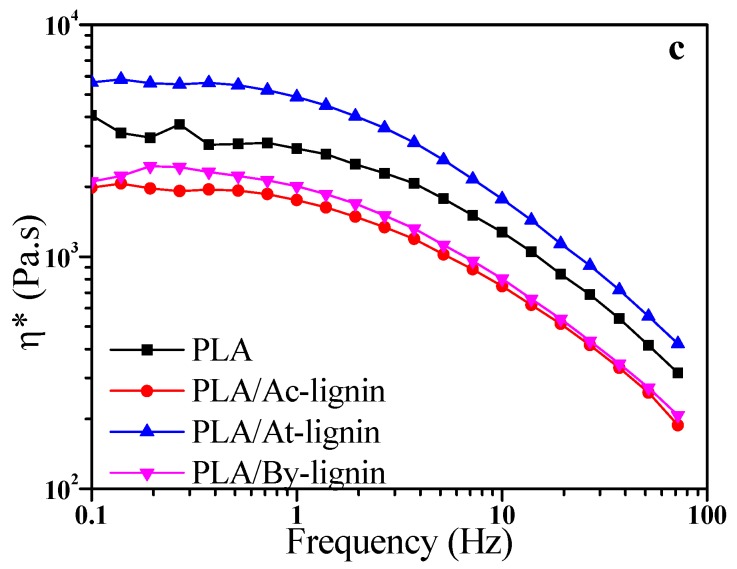
Frequency ramp sweep curves for pure PLA, PLA/Ac-lignin, PLA/At-lignin, and PLA/By-lignin biocomposites: (**a**) Storage modulus (*G*′), (**b**) loss modulus (*G″*), (**c**) complex viscosity (η*).

Figure 16 exhibits the curves of storage modulus (*G*′), loss modulus (*G″*), and complex viscosity (η*) of pure PLA and PLA/lignin with different frequency at 170 °C. The η* values for all samples decreased obviously with increasing the frequency, which means that the pure PLA and PLA/lignin biocomposites were typical pseudoplastic fluid. Both the values of *G*′ and *G″* also increased at higher frequency according to Figure 16a,b, which corresponds to the linear viscoelasticity theory of polymer. When frequency is low, the variation of shear force is relatively slow so that the movement of the polymer molecular chain is able to catch up with the variation of stresses. In this condition, the value of *G″* was slightly higher than that of *G*′ and the materials showed a viscosity. At higher frequency, the value of *G*′ increased more easily and became higher than *G″*. The movement of the polymer molecular chain was gradually not able to respond to the stress variations, which means that the materials showed an elasticity instead of viscosity. With further increasing frequency (>10 Hz), the increasing rate of *G″* became much lower and the value of *G″* kept stable. In addition, the rate of *G*′/*G″* also increased; therefore, the materials became more elastic [50].

According to Figure 16, the values of *G*′, *G″*, and η* of PLA/At-lignin were much higher than pure PLA while those of PLA/Ac-lignin and PLA/By-lignin became lower. The possible reason is due to the relatively worse coefficient of friction and interface effect of the Ac-lignin and By-lignin. Additionally, the difference in η* of the two biocomposites became negligible at high frequencies. The much higher η* of PLA/At-lignin proved that miscibility, interfacial compatibility, and interface interaction between At-lignin and PLA were better than those of the other two biocomposites, which were described in previous subsections.

**Figure 17:**
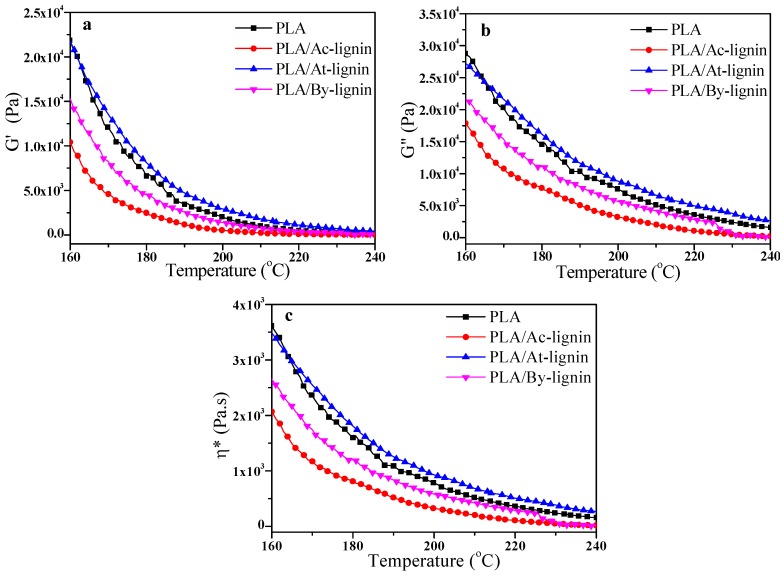
Temperature ramp sweep curves for pure PLA, PLA/Ac-lignin, PLA/At-lignin, and PLA/By-lignin biocomposites: (**a**) Storage modulus (*G*′), (**b**) loss modulus (*G″*), (**c**) complex viscosity (η*).

In view of the temperature ramp sweep results of different rigidity aromatic lignin and PLA biocomposites shown in Figure 17, the formation of the interfacial interaction is depicted. As indicated in Figure 17, the values of *G*′, *G″*, and η* of all samples decreased dramatically with increasing the temperature from 160 °C to 180 °C. Then a comparatively slower decline from 180 °C to 220 °C was seen, due to the complete melting of PLA. The decrease shows that the biocomposites turned to viscous state gradually. In this range of temperature, the value of *G″* was still higher than that of *G*′, values at a same temperature, which means that the viscous flow of PLA predominated rather than elastic deformation.

Obviously, it was exhibited that PLA/At-lignin biocomposites took possession of higher *G*′, *G″*, and η* values than pure PLA, PLA/Ac-lignin, and PLA/By-lignin biocomposites across the whole temperature range. This was also due to the formation of hydrogen bonding between PLA and At-lignin, as same as the results in frequency sweep.

### 3.6. Tensile Properties of the Biocomposites

**Figure 18:**
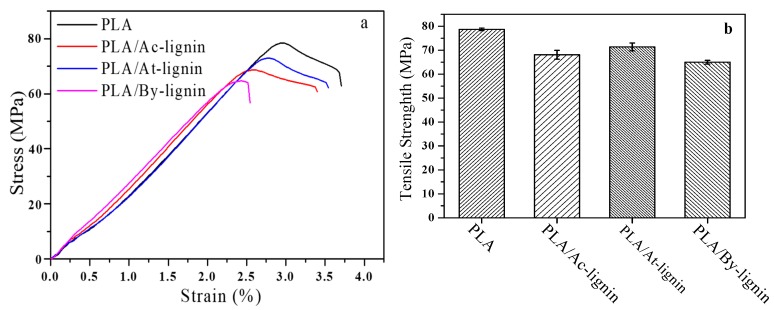
Tensile stress–strain curves (**a**) and tensile strength statistical histogram (**b**) of pure PLA and PLA/lignin biocomposites.

The tensile stress–strain curves and the histogram of the tensile strength in accordance with pure PLA and PLA/lignin biocomposites are presented in Figure 18. The PLA/Ac-lignin, PLA/At-lignin, and PLA/By-lignin biocomposites exhibited a decrease in tensile strength compared with pure PLA, owing to recombination of different lignin and poor dispersion and distribution of lignin particles in micron size inside the polymer matrix of PLA/lignin biocomposites [51,52]. The defection and stress concentration of the composites were key factors to illustrate the decrement of tensile strength [53]. The results indicated that the existence of lignin particles, which hindered the formation of continuous PLA with long range, resulting in particles to agglomerate at the interface with the neat PLA. This will decrease the tensile properties. The decrease in tensile strength for PLA/At-lignin biocomposites was less than PLA/Ac-lignin and PLA/By-lignin biocomposites. A point to note is that PLA/At-lignin biocomposites displayed the highest tensile strength among three biocomposites. The less drop in tensile strength for PLA/At-lignin biocomposites was probably attributed to the better interfacial characteristics due to the hydrogen bonding between acetoacetate groups and PLA in view of PLA/Ac-lignin and PLA/By-lignin biocomposites. In addition, the interface compatibility between At-lignin and PLA was better than the others, which led to increase the effective interfacial adhesion between lignin and PLA matrix. Consequently, PLA/At-lignin biocomposites can maximize the maintenance of the tensile strength of PLA compared to PLA/Ac-lignin and PLA/By-lignin biocomposites. The reason is that Ac-lignin and By-lignin particles in polymer matrix tend to agglomerate together to form larger lignin agglomerated particles. This can cause the agglomerated lignin particles in PLA matrix to act as a stress concentration point when subjected to tensile extension, where the stress applied on the PLA matrix was unable to be effectively transferred between the reinforcing lignin particles and PLA matrix. Thus, the addition of Ac-lignin and By-lignin particles in PLA matrix did not provide the reinforcement effect to PLA matrix [54,55]. The transition in the phase morphology of different lignin seemed to have significant influence on the mechanical properties. The transition in the morphologies from continuous PLA to continuous lignin phase with the change of lignin structure resulted in exhibiting a decreasing trend in tensile strength compared with pure PLA.

## 4. Conclusions

Significantly, it is worthwhile mentioning that Ac-lignin has been used to synthesize At-lignin and By-lignin by proper chemical modification. Ac-lignin, At-lignin, and By-lignin present different particle size and shape, which plays a significant role on compatibility and consistency between the lignin and PLA matrix. PLA/At-lignin biocomposites embrace better interfacial compatibility, which is ascribed to the hydrogen bonding interaction. As a consequence, PLA/At-lignin biocomposites took possession of higher tensile strength compared with other composites, resulting in the improvement in the compatibility between the PLA matrix and lignin filler. PLA/At-lignin biocomposites embraced higher storage modulus compared with PLA/Ac-lignin and PLA/By-lignin biocomposites as a consequence of relatively outstanding matrix-filler interface assigned to mobility of molecular chains. It was concluded from the DSC results that the *T*_g_ values of three biocomposites were changed slightly for different lignin derivatives. In addition, the *T*_g_ values of PLA/At-lignin and PLA/By-lignin biocomposites were slightly higher than of that of PLA/Ac-lignin biocomposites. This revealed that PLA/At-lignin and PLA/By-lignin biocomposites present higher thermal stability than PLA/Ac-lignin biocomposites, which was also confirmed further by the TGA results.

## Figures and Tables

**Table 1:** Composition of PLA/Ac-lignin, PLA/At-lignin, and PLA/By-lignin biocomposites.

Designation	PLA (wt %)	Ac-lignin (wt %)	At-lignin (wt %)	By-lignin (wt %)
Pure PLA	100	0	0	0
PLA/Ac-lignin	95	5	0	0
PLA/At-lignin	95	0	5	0
PLA/By-lignin	95	0	0	5

**Table 2:** The DSC data of PLA/lignin biocomposites.

Entry	*T*_g_ (°C)	*T*_m_ (°C)	Δ*H*_m_ (J/g)
PLA	62.3	150.0	3.3
PLA/Ac-lignin	61.2	151.9	18.6
PLA/At-lignin	61.6	152.7	12.0
PLA/By-lignin	61.7	153.8	4.0

**Table 3:** Characteristic thermal degradation temperatures (*T*_max_) and wt % of char residues from TGA curves.

Samples	*T*_5%_ (°C)	*T*_max_ (°C)	Char Residues at 500 °C (wt %)
PLA	389.4	423.5	0.5
PLA/Ac-lignin	357.5	407.7	3.4
PLA/At-lignin	371.5	413.4	2.4
PLA/By-lignin	374.9	413.2	3.9
